# Supplementary material for: Associations between Lifestyle Factors and Vitamin E Metabolites in the General Population
Source: Antioxidants (Basel). 2020 Dec 15;9(12):1280. doi: 10.3390/antiox9121280 (PMC7765431; doi:10.3390/antiox9121280)
Supplement: Supplementary file 1 [file antioxidants-09-01280-s001.pdf]

Supplementary tables

Table S1 Associations between alcohol use and different measurements of vitamin E activity.

Lifestyle factors		α -TOH		Total TLHQ		Total CEHC		Ratio TLHQ/CEHC	
	N =	% change	95% CI	% change	95% CI	% change	95% CI	% change	95% CI
Alcohol use in g/day									
1.0–2.49 g/day									
All	132	Ref	Ref	Ref	Ref	Ref	Ref	Ref	Ref
Men	40	Ref	Ref	Ref	Ref	Ref	Ref	Ref	Ref
Women	92	Ref	Ref	Ref	Ref	Ref	Ref	Ref	Ref
2.5–9.2 g/day									
All	133	−2	−5 to 2	−8	−19 to 6	−1	−16 to 16	−8	−18 to 4
Male	48	−2	−8 to 3	−4	−24 to 19	13	−14 to 49	−16	−33 to 5
Female	85	−2	−6 to 3	−10	−24 to 7	−9	−25 to 12	−2	−15 to 13
9.2–21.5 g/day									
All	133	−0	−3 to 4	−9	−21 to 4	2	−14 to 20	−12	−22 to 0
Male	63	−1	−6 to 5	2	−17 to 26	16	−10 to 51	−13	−29 to 8
Female	70	1	−4 to 6	−16	−30 to 2	−6	−25 to 18	−11	−24 to 5
21.5–171.6 g/day									
All	132	3	−1 to 7	−17	−28 to −4	−4	−20 to 14	−13	−24 to −1
Male	95	3	−3 to 8	−7	−24 to 14	7	−17 to 39	−13	−29 to 17
Female	37	2	−4 to 8	−27	−42 to −9	−11	−32 to 16	−19	−33 to −2

Results are derived from linear regression model adjusted for age, sex (in analysis for all participants only), BMI and smoking with 95% confidence interval (CI) and presented as percentage difference compared with the reference group. All analysis done with α -TOH were adjusted for LDL levels. α -TOH, α -Tocopherol; TLHQ, tocopheronolactone hydroquinone; CEHC, carboxymethyl-hydroxychroman.

Table S2 Associations between sleep quality, physical activity and different measurements of vitamin E activity.

Lifestyle factors		α-TOH		Total TLHQ		Total CEHC		Ratio TLHQ/CEHC	
	N =	% change	95% CI	% change	95% CI	% change	95% CI	% change	95% CI
Sleep quality									
Good Quality									
All	288	Ref	Ref	Ref	Ref	Ref	Ref	Ref	Ref
Male	158	Ref	Ref	Ref	Ref	Ref	Ref	Ref	Ref
Female	130	Ref	Ref	Ref	Ref	Ref	Ref	Ref	Ref
Poor Quality									
All	205	3	1 to 6	0	-10 to 10	-0	-12 to 13	1	-9 to 9
Male	76	5	1 to 9	6	-8 to 23	-2	-18 to 17	9	-6 to 25
Female	129	2	-1 to 6	-6	-19 to 8	-0	-15 to 18	7	-17 to 4
PA in MET-h/wk									
PA 0–16.3									
All	129	Ref	Ref	Ref	Ref	Ref	Ref	Ref	Ref
Male	61	Ref	Ref	Ref	Ref	Ref	Ref	Ref	Ref
Female	68	Ref	Ref	Ref	Ref	Ref	Ref	Ref	Ref
PA 16.5–29.8									
All	130	1	−2 to 5	−7	−18 to 7	11	−5 to 31	−17	−26 to −6
Male	59	−1	−5 to 4	−7	−23 to 12	18	−7 to 50	−22	−35 to −6
Female	71	3	−2 to 9	−8	−24 to 11	5	−16 to 31	−13	−26 to 1
PA 30.0–49.5									
All	132	−1	−4 to 3	−11	−22 to 2	0	−14 to 18	−12	−22 to −1
Male	60	−2	−7 to 3	−11	−26 to 8	1	−19 to 28	−12	−27 to 5
Female	72	−0	−5 to 5	−12	−28 to 7	−3	−23 to 21	−11	−24 to 5

PA 49.8–242.5									
All	130	−2	−6 to 1	−5	−17 to 14	4	−11 to 23	−9	−19 to 3
Male	63	−5	−10 to −0	−6	−22 to 14	15	−9 to 45	−17	−31 to −1
Female	67	−0	−5 to 6	−6	23 to 14	-6	−25 to 18	−2	−16 to 15

Results are derived from linear regression model adjusted for age, sex (in analysis for all participants only), BMI or TBF with 95% confidence interval (CI) and presented as percentage difference compared with the reference group. Analysis on sleep quality was additionally adjusted for smoking. PA was additionally adjusted for smoking and alcohol. All analysis done with α-TOH were adjusted for LDL levels. PA, Physical Activity; α-TOH, α-tocopherol; TLHQ, tocopheronolactone hydroquinone; CEHC, carboxymethyl-hydroxychroman.

Table S3 Associations between smoking, diet quality and different measurements of vitamin E activity.

**Lifestyle factors**		**α-TOH**		**Total TLHQ**		**Total CEHC**		**Ratio TLHQ/CEHC**	
	**N =**	**% change**	**95% CI**	**% change**	**95% CI**	**% change**	**95% CI**	**% change**	**95% CI**
Smoking									
Nonsmokers									
All	216	Ref	Ref	Ref	Ref	Ref	Ref	Ref	Ref
Male	92	Ref	Ref	Ref	Ref	Ref	Ref	Ref	Ref
Female	124	Ref	Ref	Ref	Ref	Ref	Ref	Ref	Ref
Former smokers									
All	255	−1	−4 to 2	4	−6 to 15	2	−10 to 15	2	−7 to 12
Male	124	−0	−4 to 4	2	−12 to 18	−6	−22 to 13	8	−7 to 25
Female	131	−2	−6 to 2	6	−8 to 23	8	−9 to 28	−1	−12 to 11
Current smokers									
All	59	−4	−8 to 0	−11	−24 to 5	−32	−44 to −18	32	14 to 53
Male	30	−4	−9 to 2	2	−18 to 28	−27	−45 to −3	39	12 to 74
Female	29	−5	−11 to 1	−21	−38 to 0	−39	−54 to −19	28	6 to 56
DHDI									
Score 25.4-61.5									

All	132	Ref	Ref	Ref	Ref	Ref	Ref	Ref	Ref
Male	75	Ref	Ref	Ref	Ref	Ref	Ref	Ref	Ref
Female	57	Ref	Ref	Ref	Ref	Ref	Ref	Ref	Ref
Score 61.9–72.5									
All	133	−2	−3 to 4	0	−13 to 15	6	−10 to 25	−7	−18 to 4
Male	72	2	−3 to 7	2	−15 to 22	7	−14 to 34	−6	−21 to 12
Female	61	−2	−7 to 4	2	−17 to 26	8	−16 to 38	−6	21 to 12
Score 72.5–81.5									
All	133	1	−2 to 5	13	−2 to 29	6	−10 to 25	5	−7 to 18
Male	62	−1	−5 to 4	6	−12 to 27	0	−20 to 26	4	−13 to 24
Female	71	3	−2 to 9	22	−1 to 49	12	−12 to 43	7	−10 to 26
Score 81.6–117.8									
All	132	3	−1 to 7	12	−3 to 29	10	−7 to 31	−1	−13 to 12
Male	37	2	−3 to 8	12	−10 to 39	18	−10 to 55	8	−25 to 14
Female	95	3	−2 to 9	15	− to 40	9	−13 to 37	3	−12 to 21

Results are derived from linear regression model adjusted for age, sex (in analysis for all participants only), BMI or TBF with 95% confidence interval (CI) and presented as percentage difference compared with the reference group. Analysis on smoking were additionally adjusted for alcohol use. Diet quality,was additionally adjusted for smoking. All analysis done with α -TOH were adjusted for LDL levels. DHDI, Dutch Healthy Diet Index; α -TOH, α -tocopherol; TLHQ, tocopheronolactone hydroquinone; CEHC, carboxymethyl-hydroxychroman.

Table S4 Associations between smoking intensity in current smokers and different measurements of vitamin E activity.

**Smoking intensity in current smokers**		**α -TOH**		**Total TLHQ**		**Total CEHC**		**Ratio TLHQ/CEHC**	
Smoking packyears 0.6-16.67	**N =**	**% change**	**95% CI**	**% change**	**95% CI**	**% change**	**95% CI**	**% change**	**95% CI**
All									
Male	29	−3	−9 to 3	−7	−26 to 17	−25	−43 to −2	24	2 to 52
Female	11	−1	−9 to 8	21	−14 to 70	−5	−37 to 44	27	−10 to 78

Smoking packyears 18.1–100.5	18	−5	−12 to 4	−18	−40 to 12	−35	−55 to −6	26	−3 to 62
All									
Male	30	−9	−15 to -3	−16	−34 to 6	−46	−59 to −28	55	25 to 93
Female	19	−10	−17 to -3	−13	−37 to 20	−50	−66 to −26	73	27 to 137
	11	−9	−18 to 2	−27	−50 to 7	−45	−65 to−14	33	−3 to 83

Results are derived from linear regression model adjusted for age, sex (in analysis for all participants only), BMI or TBF and alcohol use with 95% confidence interval (CI) and presented as percentage difference compared with the reference group. All analysis done with α -TOH were adjusted for LDL levels. α -TOH, α -tocopherol; TLHQ, tocopheronolactone hydroquinone; CEHC, carboxymethyl-hydroxychroman.
